# Bacteria and poisonous plants were the primary causative hazards of foodborne disease outbreak: a seven-year survey from Guangxi, South China

**DOI:** 10.1186/s12889-018-5429-2

**Published:** 2018-04-18

**Authors:** Yongqiang Li, Yaling Huang, Jijun Yang, Zhanhua Liu, Yanning Li, Xueting Yao, Bo Wei, Zhenzhu Tang, Shidong Chen, Decheng Liu, Zhen Hu, Junjun Liu, Zenghui Meng, Shaofa Nie, Xiaobo Yang

**Affiliations:** 1 Department of Epidemiology and Health Statistics, School of Public Health of Huazhong University of Science and Technology, Wuhan, Hubei China; 2 Guangxi Food and Drug Administration, Nanning, Guangxi China; 3 Department of Epidemiology and Health Statistics, School of Public Health of Guangxi Medical University, Nanning, Guangxi China; 4 Center for Genomic and Personalized Medicine, Guangxi Medical University, Nanning, Guangxi China; 5 Guangxi Collaborative Innovation Center for Genomic and Personalized Medicine, Nanning, Guangxi China; 6 Department of Occupational Health and Environmental Health, School of Public Health of Guangxi Medical University, Shuangyong road, Nanning, 530021 Guangxi China; 7 Guangxi Zhuang Autonomous Region Center for Disease Control and Prevention, Nanning, Guangxi China; 8 School of Information and Management of Guangxi Medical University, Nanning, Guangxi China; 9 Guangxi Zhuang Autonomous Region Health and Family Planning Commission, Nanning, Guangxi China

**Keywords:** Foodborne diseases, Foodborne disease outbreaks, Epidemiology

## Abstract

**Background:**

Foodborne diseases are a worldwide public health problem. However, data regarding epidemiological characteristics are still lacking in China. We aimed to analyze the characteristics of foodborne diseases outbreak from 2010 to 2016 in Guangxi, South China.

**Methods:**

A foodborne disease outbreak is the occurrence of two or more cases of a similar foodborne disease resulting from the ingestion of a common food. All data are obtained from reports in the Public Health Emergency Report and Management Information System of the China Information System for Disease Control and Prevention, and also from special investigation reports from Guangxi province.

**Results:**

A total of 138 foodborne diseases outbreak occurred in Guangxi in the past 7 years, leading to 3348 cases and 46 deaths. Foodborne disease outbreaks mainly occurred in the second and fourth quarters, and schools and private homes were the most common sites. Ingesting toxic food by mistake, improper cooking and cross contamination were the main routes of poisoning which caused 2169 (64.78%) cases and 37 (80.43%) deaths. Bacteria (62 outbreaks, 44.93%) and poisonous plants (46 outbreaks, 33.33%) were the main etiologies of foodborne diseases in our study. In particular, poisonous plants were the main cause of deaths involved in the foodborne disease outbreaks (26 outbreaks, 56.52%).

**Conclusions:**

Bacteria and poisonous plants were the primary causative hazard of foodborne diseases. Some specific measures are needed for ongoing prevention and control against the occurrence of foodborne diseases.

**Electronic supplementary material:**

The online version of this article (10.1186/s12889-018-5429-2) contains supplementary material, which is available to authorized users.

## Background

Foodborne diseases have been an issue for all societies and are a continuing public health problem. Annually, one-third of people worldwide are infected by foodborne pathogens [1]. The Foodborne Disease Burden Epidemiology Reference Group (FERG), established by the World Health Organization (WHO) in 2007, estimated that 31 foodborne hazards caused 600 million foodborne illnesses and 420,000 deaths in 2010 [2]. In addition, the global burden of foodborne disease by these 31 hazards was 33 million disability-adjusted life years (DALYs) in 2010. Therefore, more attention should be focused on foodborne disease.

In developing countries, such as China, food safety remains a social health problem, playing a crucial role in public health [3], and it is directly related to social stability. From 2001 to 2010, 5021 outbreaks of foodborne disease were reported in China, causing 140,101 illnesses and 1427 deaths [4]. From 2004 to 2013, the mortality rate of foodborne diseases was 1.9% in China [5]. Major sources of foodborne diseases in China include pathogenic microorganisms, toxic insects/food products and plants entering the food supply, and chemical contamination [6]. A total of 371 outbreaks related to bacterial foodborne diseases were reported in China between 2008 and 2010, and 20,062 cases and 41 deaths were involved [7]. Since 2000, a total number of 52 cases of mushroom poisoning due to *Amanita exitialis* have been reported, causing 20 deadths [8].

Foodborne diseases and diseases potentially transmitted by food are investigated by the China Food and Drug Administration (CFDA) in China. The CFDA conducts surveillance in the Public Health Emergency Report and Management Information System of the China Information System for Disease Control and Prevention. The CFDA has monitored illness trends, detected outbreaks, provided information about preventative measures, evaluated the efficacy of intervention efforts and carried out work based on the Food Safety Law since 2009.

Although the surveillance system for foodborne diseases and the law on food safety have been effective in China, foodborne diseases remain a severe public health problem, and few studies or data analyses have focused on it. In this paper, we investigated the prevalence and epidemiological characteristics of foodborne disease outbreaks from 2010 to 2016 in Guangxi, and provided evidence for the ongoing prevention and control against foodborne diseases.

## Methods

### Definitions

Foodborne disease is defined as any disease of an infectious or toxic nature caused by consumption of food [9], with clinical symptoms, such as nausea, vomiting, abdominal pain, diarrhea, other gastrointestinal symptoms or nerve symptoms of poisoning. Food poisoning cases are reported on the basis of assessment by physicians at the hospital and confirmed by epidemiologists from local Centers for Disease Control (CDC). A foodborne disease outbreak is the occurrence of two or more cases of similar foodborne disease resulting from the ingestion of a common food. In this study, we analyzed only outbreaks that were caused by diseases of toxic nature (vs. infectious).

We divided a year into four quarters: the first quarter (January, February, March), the second quarter (April, May, June), the third quarter (July, August, September) and the fourth quarter (October, November, December).

### Data collection

The official statistics regarding foodborne disease outbreaks reported between 2010 and 2016 in Guangxi were downloaded from the information reported in the Public Health Emergency Report and Management Information System of the China Information System for Disease Control and Prevention, and were summarized from foodborne disease special investigation reports around Guangxi. A single outbreak was reported on a food hygiene incident report card. The card contains the number of cases and deaths, onset date, outbreak areas and sites, implicated foods, contributing factors and causative hazards. Implicated foods were categorized as grains, meat and poultry, seafood, eggs, dairy products, cakes and bakery products, vegetables, toxic insects/food products, poisonous plants and others, which were not confirmed. Contributing factors include material contamination and deterioration, equipment contamination, handling contamination, improper cooking, cross contamination, long preparation time, ingesting poisonous food by mistake and deliberate poisoning. Causative hazards, such as bacteria, poisonous plants, chemical agents and toxic insects/food products, were confirmed by laboratory tests and epidemiological investigation. Factors which failed to be detected by existing laboratory conditions but confirmed by epidemiological investigation were classified as “unconfirmed”. The outbreak areas were categorized as village, town and city. The outbreak sites were categorized as canteens, private homes, schools, restaurants and delicatessens. All the laboratory test methods were performed in accordance with national methods of food hygienic analysis.

### Statistical analysis

All analyses were performed using SPSS statistical program, version 16.0. A chi-square test was used to compare differences in death among quarters and areas, and causative hazard. The Bonferroni test was used for pairwise comparison of death. A nominal two-sided *P* value less than 0.05 was considered to indicate statistical significance. A two-sided *P*´ value less than $\frac{0.05}{C_k^2}$ (k represents the number of groups) was considered to indicate statistical significance in the Bonferroni test. All tables and figures were devised using Microsoft Excel software, version 2007 and GraphPad Prism, version 5.01, respectively.

## Results

### General characteristics

From 2010 to 2016, 138 foodborne disease outbreaks occurred, with a mean of 19.7 (ranging from 11 to 29) foodborne disease outbreaks being reported annually (Table 1). A total of 3348 cases and 46 deaths were involved in foodborne disease outbreak during those 7 years, with 24.26 cases per outbreak. The morbidity of foodborne disease outbreak ranged from 0.63 to 1.59 cases per 100,000 people, and the case fatality rates ranged from 0.32 to 2.48%.

**Table 1:** Number of outbreaks, cases and deaths by year, Guangxi, South China, 2010–2016

Year	No.(%) of outbreaks	No.(%) of cases	No.(%) of deaths	Morbidity ^a^ (1/100000)	Case fatality rate (%)
2010	21 (15.22)	444 (13.26)	11 (23.91)	0.96	2.48
2011	29 (21.01)	738 (22.04)	13 (28.26)	1.59	1.76
2012	16 (11.59)	343 (10.24)	6 (13.04)	0.73	1.75
2013	11 (7.97)	296 (8.84)	3 (6.52)	0.63	1.01
2014	21 (15.22)	625 (18.67)	2 (4.35)	1.31	0.32
2015	21 (15.22)	409 (12.22)	8 (17.39)	0.85	1.96
2016	19 (13.77)	493 (14.73)	3 (6.52)	1.03	0.61
total	138	3348	46	1.02	1.37

^a^The morbidity were calculated basing on the Guangxi population reported in Guangxi Statistical Year book

### Time distribution

The lowest number of outbreaks (28 of 138, 20.29%) and cases (704 of 3348, 21.03%) occurred in the first quarter compared to the other three quarters (Table 2). The case fatality rate in the third quarter (0.42%) was the lowest, while it was the highest in the fourth quarter (1.87%). The outbreaks mainly occurred in January, April, November, October, May and July, and in February the number of cases was the lowest during months investigated. In addition, the case fatality was the highest in May (Additional file 1). A significant difference was found for death between the second and third quarters, and between the third and fourth quarters (*p* = 0.018).

**Table 2:** Number of outbreaks, cases and deaths of quarters, areas and causative hazard, Guangxi, South China, 2010–2016

	No.(%) of outbreaks	No.(%) of cases	No.(%) of deaths	Case fatality rate (%)	*p* value ^a^
Quarters ^b^					0.008
First	28 (20.29)	704 (21.03)	9 (19.57)	1.28	
Second	39 (28.26)	963 (28.76)	19 (41.30)	1.97	
Third	31 (22.46)	931 (27.81)	4 (8.70)	0.43	
Fourth	40 (28.99)	750 (22.40)	14 (30.43)	1.87	
Areas ^c^					0.001
Village	50 (36.23)	1202 (35.90)	20 (43.48)	1.66	
Town	50 (36.23)	1158 (34.59)	23 (50.00)	1.99	
City	38 (27.54)	988 (29.51)	2 (6.52)	0.30	
Causative hazard ^d^					< 0.001
Bacteria	62 (44.93)	2123 (63.41)	6 (13.04)	0.28	
Chemical agents	9 (6.52)	131 (3.91)	8 (17.39)	6.11	
Toxic insects/food products	3 (2.17)	15 (0.45)	3 (6.52)	20.00	
Poisonous plants	46 (33.33)	711 (21.24)	26 (56.52)	3.66	
Factors unconfirmed	18 (13.04)	368 (10.99)	3 (6.52)	0.82	
Total	138	3348	46	1.02	

^a^Differences in death among quarters, areas, and causative hazard were compared by Chi-Square test. ^b^ Differences in death were significant in the comparison of the second and the third quarter, and the comparison the third and the fourth quarter (*p* = 0.018). ^c^ Differences in death were significant in all areas pairwise comparison (*p* = 0.017) except comparison of village and town (*p* = .0558). ^d^ Differences in death were significant in all causative hazard pairwise comparison (*p* = 0.018) except comparison of chemical agents and toxic insects/food products (*p* = 0.053), and chemical agents and poisonous plants (*p* = 0.191)

### Distribution of areas and sites

The outbreaks were likely to occur in villages and towns. As shown in Table 2, the proportion of outbreaks and cases in villages and towns were higher than that in cities. As for the occurring sites, the places of outbreaks were mainly located in schools (59 of 138, 42.75%) and private homes (45 of 138, 32.61%). The cases tended to be concentrated in schools (1600 of 3348, 47.79%), whereas the deaths mainly occurred in private homes (36 of 46, 78.26%) (Table 3).

**Table 3:** Number of outbreaks, cases and deaths by site, Guangxi, South China, 2010–2016

Site	No.(%) of outbreaks (*n* = 138)	No.(%) of cases (*n* = 3348)	No.(%) of deaths (*n* = 46)	Case fatality rate (%)
Canteens	7 (5.07)	106 (3.17)	0 (0.00)	0.00
Private homes	45 (32.61)	814 (24.31)	36 (78.26)	4.42
Schools	59 (42.75)	1600 (47.79)	0 (0.00)	0.00
Restaurants	10 (7.25)	407 (12.16)	0 (0.00)	0.00
Delicatessens	9 (6.52)	299 (8.93)	0 (0.00)	0.00
Others ^a^	8 (5.80)	122 (3.64)	10 (21.74)	8.20

^a^Other sites include construction sites and field

### Categories of poisoning food and transmission routes

Up to ten kinds of food were found to be related to foodborne disease outbreaks. The top three were poisonous plants (46, 33.33%), mixed food (39, 28.26%) as well as meat and poultry (15, 10.87%). In particular, poisonous plants were the main cause of death involved in the foodborne disease outbreaks (26, 56.52%) (Table 4). The transmission routes of foodborne disease outbreaks were diverse. We found that ingesting poisonous food by mistake, improper cooking methods and cross contamination were the main transmission routes, causing 2169 (64.78%) infection cases and 37 (80.43%) deaths. Ingesting poisonous food by mistake was the main cause of death (35 of 46, 76.09%), and deliberate poisoning caused a high case fatality rate (50.00%) (Table 5).

**Table 4:** Number of outbreaks, cases and deaths by different kinds of food, Guangxi, South China, 2010–2016

Food	No.(%) of outbreaks (*n* = 138)	No.(%) of cases (*n* = 3348)	No.(%) of deaths (*n* = 46)	Case fatality rate (%)
Grains	8 (5.80)	164 (4.90)	6 (13.04)	3.66
Meat and poultry	15 (10.87)	513 (15.32)	0 (0.00)	0.00
Seafood	5 (3.62)	141 (4.21)	0 (0.00)	0.00
Eggs	2 (1.45)	54 (1.61)	0 (0.00)	0.00
Dairy products	1 (0.72)	15 (0.45)	0 (0.00)	0.00
Cakes and bakery products	4 (2.90)	94 (2.81)	0 (0.00)	0.00
Vegetables	2 (1.45)	51 (1.52)	0 (0.00)	0.00
Toxic insects/food products	3 (2.17)	15 (0.45)	3 (6.52)	20.00
Poisonous plants	46 (33.33)	711 (21.24)	26 (56.52)	3.66
Mixed food ^a^	39 (28.26)	1238 (36.98)	4 (8.70)	0.32
Others ^b^	13 (9.42)	352 (10.51)	7 (15.22)	1.99

^a^Mixed Food was identified as at least two kinds of food involved in a food poisoning incident

^b^Other kind of food include frog and the food we cannot confirm

**Table 5:** Number of outbreaks, cases and deaths by different ways of poisoning, Guangxi, South China, 2010–2016

Contributing factors	No.(%) of outbreaks (*n* = 98)	No.(%) of cases (*n* = 2446)	No.(%) of deaths (*n* = 35)	Case fatality rate (%)
Material contamination and deterioration	14 (10.14)	317 (9.47)	6 (13.04)	1.89
Equipment contamination	2 (1.45)	99 (2.96)	0 (0.00)	0.00
Handlers contaminating	2 (1.45)	118 (3.52)	0 (0.00)	0.00
Improper cooking	27 (19.57)	849 (25.36)	2 (4.35)	0.24
Cross contamination	20 (14.49)	747 (22.31)	0 (0.00)	0.00
Long preparation time	9 (6.52)	232 (6.93)	1 (2.17)	0.43
Ingesting poisonous food by mistake	45 (32.61)	573 (17.11)	35 (76.09)	6.11
Deliberate poisoning	1 (0.72)	2 (0.06)	1 (2.17)	50.00
Others ^a^	18 (13.04)	411 (13.04)	1 (2.17)	0.24

^a^Other sources of poisoning include using the contaminated water and the sources we cannot identify

### Causative hazard

**Fig. 1:**
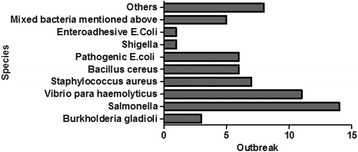
Number of outbreaks of the bacterial causative hazard, Guangxi, South China, 2010–2016

**Fig. 2:**
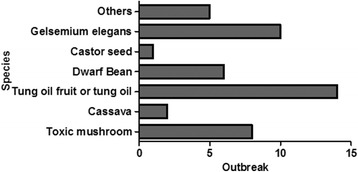
Number of outbreaks of the toxic plant causative hazard, Guangxi, South China, 2010–2016

The causative hazards were classified into bacteria, poisonous plants, chemical agents and toxic insects/food products. Bacteria (62 of 138, 44.93%) and poisonous plants (46, 33.33%) were the first and second factors for foodborne disease outbreaks, respectively (Table 2). In addition, the main species of bacteria contributing to foodborne disease outbreaks were *Salmonella, Vibrio parahaemolyticus* and *Staphylococcus aureus* (Fig. 1). Tung oil fruit or tung oil, Gelsemium elegans and toxic mushroom were the primary sources of poisoning from poisonous plants (Fig. 2). Contaminations by *Burkholderia gladioli* (6 of 46, 13.04%), toxic mushroom (5 of 46, 10.87%) and Gelsemium elegans (17 of 46, 36.96%) were the main factors for deaths (Table 6). Notably, there were 30 outbreaks in which the causative hazard could not be found, involving 719 cases and 9 deaths. Significant differences were found in the deaths of the five causative hazards (*p <* 0.001), and deaths caused by toxic insects/food products was higher than those caused by bacteria and poisonous plants.

**Table 6:** Number of cases and deaths by different Causative hazard, Guangxi, South China, 2010–2016

Causative hazard	No.(%) of cases	No.(%) of deaths
Bacteria	2123 (63.41)	6 (13.04)
*Burkholderia gladioli*	11 (0.33)	6 (13.04)
*Salmonella*	735 (21.95)	0 (0.00)
*Vibrio para haemolyticus*	375 (11.20)	0 (0.00)
*Staphylococcus aureus*	128 (3.82)	0 (0.00)
*Bacillus cereus*	113 (3.38)	0 (0.00)
*Pathogenic E.coli*	108 (3.23)	0 (0.00)
*Shigella*	81 (2.42)	0 (0.00)
*Enteroadhesive E.Coli*	25 (0.75)	0 (0.00)
Mixed bacteria mentioned above	284 (8.48)	0 (0.00)
Others	263 (7.86)	0 (0.00)
Chemical agents	131 (3.91)	8 (17.39)
Fluoroacetamide	2 (0.06)	2 (4.35)
Tetramine	7 (0.21)	2 (4.35)
Sulfur dioxide and dehydroacetic acid	83 (2.48)	0 (0.00)
Nitrite	18 (0.54)	0 (0.00)
Others	21 (0.63)	4 (8.70)
Toxic insects/food products	15 (0.45)	3 (6.52)
Toxic honey	11 (0.33)	2 (4.35)
Mylabris cichorii	4 (0.12)	1 (2.17)
Poisonous plants	711 (21.24)	26 (56.52)
Toxic mushroom	52 (1.55)	5 (10.87)
Cassava	8 (0.24)	2 (4.35)
Tung oil fruit or tung oil	315 (9.41)	0 (0.00)
Dwarf bean	136 (4.06)	0 (0.00)
Castor seed	21 (0.63)	0 (0.00)
Gelsemium elegans	112 (3.35)	17 (36.96)
Others	67 (2.00)	2 (4.35)
Factors unconfirmed	368 (10.99)	3 (6.52)

*Abbreviation: E.coli Escherichia coli*

## Discussion

Suspicious food, contributing outbreak factors and specific causative hazard are the most crucial elements of foodborne disease outbreaks. If the linkages among them were found and appropriate measures were taken, the number of foodborne disease outbreaks could be controlled. In the present study, we investigated 138 foodborne disease outbreaks from 2010 to 2016 in Guangxi, South China; our data showed that the rates of foodborne disease outbreaks that occurred in the second and fourth quarters were higher than those in the first and third quarters. The foodborne disease outbreaks were likely to occur in schools. The main contributing factors of foodborne disease outbreaks included ingesting poisonous food by mistake, improper cooking and cross contamination, and the main causative hazard of foodborne disease outbreaks were bacteria and poisonous plants in Guangxi.

During the five-year reporting period, the number of foodborne disease outbreaks, cases and deaths decreased compared with the period from 2005 to 2009 in Guangxi [10]. This suggests that the enhanced surveillance of food poisoning had an effect on food safety since the new law was established in 2009. The new law was focused on strengthening food safety risk monitoring and assessment, and punishment for violations was more stringent than before. From 2010 to 2016, the number of foodborne disease outbreaks reported annually fluctuated substantially, and the number of outbreaks in 2014 to 2016 was higher than that in 2012 and 2013. This might be due to the reformation of the Guangxi Supervision and Management System for Food and Drugs in October 2013, which brought about a change in supervision staff and departments of catering services.

Our result was in line with previous studies, which showed that foodborne disease outbreaks were likely to occur in warm months [4, 11, 12]. Foodborne disease outbreaks were related to changes in weather [13]. The natural environment in Guangxi is subtropical monsoon weather, and this warm and humid weather is conducive to bacteria and herbal medicine growth, which is medicine made from plants and used to prevent or treat disease or promote health. Therefore, some special measures, such as strengthening public awareness and supervision of food safety, are needed to prevent foodborne disease outbreaks in these months in which foodborne disease outbreaks occurred frequently.

Li [14] found that most deaths from foodborne disease outbreaks took place in private homes between 2002 and 2011 in China. In accordance with this current study, private homes were the most severely afflicted foodborne disease outbreaks, where 78.26% of deaths occurred. This finding shows that it is extremely important for the public health authorities to teach the public population how to save themselves in case poisoning occurs at home. In addition, we also found that approximately three-quarters of outbreaks occurred in rural areas and towns, especially at schools in remote areas. Good environmental hygiene can help us to prevent many illnesses caused by infection from noxious pathogens, and can prevent noxious pathogens contaminating food. Therefore, if supervision of environmental hygiene is improved, poisoning may be controlled.

Improper practices of food handlers in hand, equipment and utensil hygiene; maintenance of temperature of food ready for consumption; cooking temperature; and thawing relates directly to foodborne disease and are the main cause of foodborne disease outbreaks [15–17]. In this study of 138 foodborne disease outbreaks, improper cooking and cross contamination were the main causes during meal preparation. Moreover, the lack of ability to identify poisonous food can easily cause poisoning, and we found that ingesting poisonous food by mistake was the primary route of contracting foodborne disease outbreaks in Guangxi. Therefore, enhancing food safety knowledge and hygiene practices of handlers and improving the knowledge of residents about toxic food may be key points for ongoing supervision.

Bacterial food poisoning is a prevalent foodborne disease worldwide [18, 19]. WHO showed that the pathogens resulting in the most foodborne cases were norovirus, *Campylobacter spp.*, *enterotoxigenic e.coli*, diarrheal disease due to non-typhoidal S. *enterica*, *and Shigella spp.* [20]. In their study found that norovirus, *enteropathogenic e.coli*, *V. cholerae,* and *Shigella spp.* were responsible for large numbers of deaths among the diarrheal diseases and *Salmonella* Typhi, hepatitis A virus, invasive infections due to non-typhoidal S. *enterica* and *Salmonella* Paratyphi A were responsible for the most deaths among the extra-intestinal enteric diseases*.* In this study, which focused only on outbreaks of foodborne disease of toxic nature (vs. infectious), we found that *Salmonella, Vibrio para haemolyticus*, *Staphylococcus aureus*, chemical agents, and tung oil fruit or tung oil were responsible for most cases. In addition, poisonous plants were the leading cause of death. We did not detect *Campylobacter* in all the samples. As is known, the relative frequency of foodborne disease depends strongly on geography, local diet, sanitary conditions and general public health, among many other factors. The main reason for the differences in foodborne hazards between WHO and our study may be a response to the local diet. In China, dairy foods, the main vehicles for *Campylobacter*, accounted less but frequently in United States [21]. In China, people generally believe that wild-harvested products, including mushrooms, have high nutritional and medicinal values and the risky behavior of collecting and consuming wild plants (such as poisonous mushroom, Gelsemium elegans and tung oil fruit) happens mostly in remote districts in household clusters [22]. Therefore, increasing public awareness about food safety of poisonous plants is an important elements of preventing and controlling future foodborne disease outbreak.

Our study showed that public awareness about food safety of poisonous plant, hand hygiene of handlers and policies about the interventions on foodborne illness were important elements preventing and controlling foodborne disease. Our findings might help in the development of a better foodborne disease monitoring system based on the characteristics of Guangxi. From this, we can list high-risk foods, including meat, poultry and poisonous plants (e.g. tung oil, tung oil fruit and Gelsemium elegans), to put forward appropriate measures for prevention and control. Tung oil is rich in eleostearic acid. If eaten by mistake, symptoms of poisoning, such as nausea, vomiting, abdominal pain, breathing difficulties, limb twitches, coma and laryngeal muscle spasm, appear within 2 h. Gelsemium elegans contains a variety of alkaloids, such as gelsemine free base, which can inhibit the respiratory center and may eventually cause respiratory center paralysis and death due to respiratory failure. Improving the training and food safety awareness of food handlers, enhancing the systems of food procurement and inspection, strengthening the disinfection of tableware and preventing improper handling and cross contamination during food preparation should also be included. Moreover, skills of investigators, such as their ability to investigate foodborne disease, identify poisonous food, and their awareness about food safety should be enhanced.

Several limitations are necessary to be noted in the current study. First, we only discussed the epidemic of foodborne disease outbreaks of Guangxi, a province of China. Guangxi is one of five ethnic minority autonomous regions in China, which includes twelve minorities and 46.82 million people in 2012. It is the only coastal autonomous region which is adjacent to the land and sea of Vietnam and the permanent venue of China-ASEAN Expo. Thus, we think it is necessary for us to discuss the epidemic characteristics of foodborne disease outbreaks and the security status of food in this autonomous region. Second, 13.04% of outbreaks were found without a confirmed etiologic agent in our study. We have made efforts to improve the collection of suspicious food after foodborne disease outbreaks occurring and the ability of food detection of our laboratory. Third, since some cases (e.g. mild acute gastro-enteritis) are self-curable and hardly recognized, reported or investigated, it is susceptible to reporting bias. Fourth, the detection ability of the present clinical laboratories in Guangxi is relatively limited, and some pathogens are perhaps not identified routinely. Finally, these findings might not be generalizable to other areas in China.

## Conclusion

The primary causative hazard of foodborne disease outbreaks in Guangxi were bacteria and poisonous plants, and the contributing factors were ingesting poisonous food by mistake, improper cooking and cross contamination. Therefore, improving the detection of bacteria in food, and the market supervision of poisonous plants and propaganda knowledge about food safety might be helpful for us to control foodborne disease outbreaks.

## Additional file

Additional file 1: Table Number of outbreaks, cases and deaths by month, Guangxi, South China, 2010–2016. (DOC 42 kb)

## Abbreviations

CDC: Centers for Disease Control
CFDA: China Food and Drug Administration
DALYs: Disability-adjusted life years
FERG: Foodborne Disease Burden Epidemiology Reference Group
WHO: World Health Organization
