# Supplementary material for: Bacteria and poisonous plants were the primary causative hazards of foodborne disease outbreak: a seven-year survey from Guangxi, South China
Source: BMC Public Health. 2018 Apr 18;18:519. doi: 10.1186/s12889-018-5429-2 (PMC5907191; doi:10.1186/s12889-018-5429-2)
Supplement: Supplementary file 1 — Table Number of outbreaks, cases and deaths by month, Guangxi, South China, 2010–2016. (DOC 42 kb) [file 12889_2018_5429_MOESM1_ESM.doc]

**S1 Table Number of outbreaks, cases and deaths by month, Guangxi, South China, 2010-2016.**

| **Month** | **No.of incidents**  **(n=138)** | **No.of cases**  **(n=3348)** | **No.of deaths**  **(n=35)** |
| --- | --- | --- | --- |
| Jan | 15 (10.87%) | 301 (8.99%) | 6 (13.04%) |
| Feb | 3 (2.17%) | 115 (3.43%) | 1 (2.17%) |
| Mar | 10 (7.25%) | 288 (8.60%) | 2 (4.35%) |
| Apr | 15 (10.87%) | 434 (12.96%) | 4 (8.70%) |
| May | 13 (9.42%) | 225 (6.72%) | 10 (21.74%) |
| Jun | 11 (7.97%) | 304 (9.08%) | 5 (10.87%) |
| Jul | 13 (9.42%) | 478 (14.28%) | 1 (2.17%) |
| Aug | 9 (6.52%) | 258 (7.71%) | 1 (2.17%) |
| Sep | 9 (6.52%) | 195 (5.82%) | 2 (5.71%) |
| Oct | 14 (10.14%) | 322 (9.62%) | 0 (0.00%) |
| Nov | 15 (10.87%) | 295 (8.81%) | 7 (15.22%) |
| Dec | 11 (7.97%) | 138 (3.97%) | 7 (15.22%) |
